# Cytotoxicity and apoptotic activities of alpha-, gamma- and delta-tocotrienol isomers on human cancer cells

**DOI:** 10.1186/1472-6882-14-469

**Published:** 2014-12-06

**Authors:** Su-Wen Lim, Hwei-San Loh, Kang-Nee Ting, Tracey D Bradshaw, Nazariah A Zeenathul

**Affiliations:** School of Biosciences, Faculty of Science, University of Nottingham Malaysia Campus, 43500 Semenyih, Malaysia; Department of Biomedical Sciences, Faculty of Science, University of Nottingham Malaysia Campus, 43500 Semenyih, Malaysia; School of Pharmacy, Faculty of Science, University of Nottingham, University Park, Nottingham, NG7 2RD UK; Department of Veterinary Pathology and Microbiology, Faculty of Veterinary Medicine, Universiti Putra Malaysia, 43400 Serdang, Malaysia

**Keywords:** Tocotrienol isomers, Cytotoxicity, Apoptosis, DNA damage, Caspase-8, Bid, Bax, Mitochondrial membrane permeability, Cytochrome *c*

## Abstract

**Background:**

Tocotrienols, especially the gamma isomer was discovered to possess cytotoxic effects associated with the induction of apoptosis in numerous cancers. Individual tocotrienol isomers are believed to induce dissimilar apoptotic mechanisms in different cancer types. This study was aimed to compare the cytotoxic potency of alpha-, gamma- and delta-tocotrienols, and to explore their resultant apoptotic mechanisms in human lung adenocarcinoma A549 and glioblastoma U87MG cells which are scarcely researched.

**Methods:**

The cytotoxic effects of alpha-, gamma- and delta-tocotrienols in both A549 and U87MG cancer cells were first determined at the cell viability and morphological aspects. DNA damage types were then identified by comet assay and flow cytometric study was carried out to support the incidence of apoptosis. The involvements of caspase-8, Bid, Bax and mitochondrial membrane permeability (MMP) in the execution of apoptosis were further expounded.

**Results:**

All tocotrienols inhibited the growth of A549 and U87MG cancer cells in a concentration- and time-dependent manner. These treated cancer cells demonstrated some hallmarks of apoptotic morphologies, apoptosis was further confirmed by cell accumulation at the pre-G_1_ stage. All tocotrienols induced only double strand DNA breaks (DSBs) and no single strand DNA breaks (SSBs) in both treated cancer cells. Activation of caspase-8 leading to increased levels of Bid and Bax as well as cytochrome *c* release attributed by the disruption of mitochondrial membrane permeability in both A549 and U87MG cells were evident.

**Conclusions:**

This study has shown that delta-tocotrienol, in all experimental approaches, possessed a higher efficacy (shorter induction period) and effectiveness (higher induction rate) in the execution of apoptosis in both A549 and U87MG cancer cells as compared to alpha- and gamma-tocotrienols. Tocotrienols in particular the delta isomer can be an alternative chemotherapeutic agent for treating lung and brain cancers.

**Electronic supplementary material:**

The online version of this article (doi:10.1186/1472-6882-14-469) contains supplementary material, which is available to authorized users.

## Background

Cancer is one of the leading causes of death globally. According to American Cancer Society [1], one in eight deaths is caused by cancer. An estimation of about 7 million people dies from cancer each year and is predicted to rise to 21.4 million new cases and 13.2 million deaths by 2030 [2].

Many previous studies have proposed that vitamin E can be a candidate for adjuvant treatment of tumorigenesis and it plays an imperative role in the prevention and occurrence of cancer [3]. Tocotrienols and tocopherols are naturally occurring isoforms of vitamin E that are found abundantly in food such as palm oil, rice bran oil, corn, oats, barley, rye and wheat [4–7]. Both isoforms have substantial antioxidant activity that may enhance their useful biological effects [6, 7]. Tocotrienols generally possess more remarkable anticancer potential than tocopherols [6, 8, 9]. And, desmethyl tocotrienols (delta- and gamma-tocotrienols) are speculated to be more bioavailable than the other isomers making desmethyl tocotrienols more favorable as potential anticancer candidates [10]. Numerous studies predominantly focusing on gamma-tocotrienol have been reported to possess antiproliferative effects against breast cancer [6, 11–13], liver cancer [14], colon cancer [7], gastric adenocarcinoma [15], prostate cancer [16–18] and lung cancer [19]. However, further studies are still required to completely explain the mechanisms for tocotrienol-induced apoptosis, attributable in part to its antiproliferative effects on cancer cells. As referred to the above-mentioned reports, tocotrienols might induce different mechanisms of action in different cancer cell types.

These interesting previous studies have captured our attention in investigating the cytotoxic effects and underlying apoptotic mechanisms of alpha-, gamma- and delta-tocotrienols on human lung adenocarcinoma (A549) and glioblastoma (U87MG) cells which are still scarcely studied.

## Methods

### Tocotrienol isomers, cell lines and culture conditions

The palm oil derived alpha-, gamma- and delta-tocotrienol isomers were extracted and supplied in palm vitamin E isomers kit by Davos Life Science Pte Ltd (Singapore). The human lung adenocarcinoma (A549), grade IV glioblastoma (U87MG) and normal lung fibroblast (MRC5) were grown and maintained at the similar conditions as previously published papers [20, 21].

### Cell viability assessment assay

Neutral red uptake assay was performed to determine the cell viability based on an established protocol [22]. A total of 5 x 10^3^ cells per well were seeded in a 96-well plate (Nunc, USA) and incubated for 24 h. All cell lines were then treated with alpha-, gamma- and delta-tocotrienols at the concentrations ranging from 1 μM to 100 μM for 24 h, 48 h and 72 h. Vinblastine was used as a positive control at the concentrations ranging from 0.011 μM to 11 μM. The percentage of cell viability was calculated using the formula: (OD_treated_/OD_untreated_ × 100%) taking into the account of vehicle control untreated cells.

### Morphological characterization by staining techniques

Morphological assessments for cellular changes, viability and apoptosis in response to each tocotrienol isomer treatment were conducted via haematoxylin and eosin (H&E), acridine orange and propidium iodide (AO&PI) as well as fluorescein diacetate and propidium iodide (FDA&PI) staining techniques. Guided by the neutral red uptake assay, cells grown in chamber slides (Lab-Tek, USA) were treated with the IC_50_ and IC_80_ values (Table 1) of each tocotrienol isomer for 24 h and 48 h. The H&E procedure was adopted as previously published papers [20, 21]. On the other hand, 10 μl of fluorescent dyes containing AO (10 μg/ml) and PI (10 μg/ml) were added onto the prepared cells. For FDA&PI staining, 2.5 μg/ml of FDA (Sigma, USA) and 1.5 μg/ml of PI (Invitrogen, USA) were prepared freshly as dyes. Treated and vehicle control cells were washed three times in PBS, stained in 200 μl of PBS containing dyes and examined immediately using Eclipse 80i research microscope with an epifluorescence illuminator attachment (Nikon, Japan). At least 200 cells in each sample were observed for features showing viability, early and late apoptosis in addition to secondary necrosis. Percentage of cells categorized at distinguished stages of apoptosis was also recorded.

**Table 1:** **IC**_**50**_ **, IC**_**80**_ **and IC**_**max**_ **concentrations of all tocotrienols used in different experiments**

Cell lines	A549			U87MG		
Concentrations (μM)	IC _50_	IC _80_	IC _max_	IC _50_	IC _80_	IC _max_
Alpha-tocotrienol	5.0	60.0	100.0	2.0	10.0	100.0
Gamma-tocotrienol	2.0	10.0	100.0	3.0	60.0	100.0
Delta-tocotrienol	2.0	10.0	100.0	1.0	10.0	100.0

Values are obtained from the concentration-response curves plotted using probit or logistic analysis in Graphpad Prism (version 5) bio-statistical software.

### Determination of DNA damage by comet assay

Single cell gel electrophoresis (SCGE or comet) assay (Trevigen, USA) was used to determine the effects of alpha-, gamma- and delta-tocotrienols (72 h treatment) on nuclear DNA damage according to previously reported protocols [20, 21]. Comets that appeared under neutral conditions provided evidence of double strand breaks (DSBs); whereas comets produced under alkaline conditions indicated single strand breaks (SSBs). The tocotrienol isomers were tested at IC_50_ concentrations in both cancer cells (Table 1) and vinblastine (0.011 μM and 11 μM) was served as a positive control. Previous reports [20, 21, 23] were followed for visual image analysis and scoring of DNA damage.

### Cell cycle phase evaluation by flow cytometry

A total of 1 × 10^6^ cells per well were seeded in a 6-well plate (Orange Scientific, Belgium) and incubated for 24 h. Cells were then treated with the IC_50_ and IC_80_ values (Table 1) of tocotrienol isomers for 24 h and 48 h. Following a standard harvest procedure, cells were resuspended in 400 μl of fluorochrome solution (0.1% Triton-X-100; 0.1% sodium citrate; 0.1 mg/ml RNase; 50 μg/ml PI) and stored in dark at 4°C overnight. Then, the samples were homogenously mixed before subjected for cell cycle analysis in a flow cytometry machine installed with EXPO32 software (Beckman Coulter Ltd, UK). Triplicates of each sample were analyzed.

### Caspase-8 activity determination assay

A total of 1 × 10^5^ cells/ml were seeded and grown in 60 mm^2^ petri dishes. Cell lines were tested at different concentrations of tocotrienol isomers (Table 1) and vinblastine at 0.011 μM and 11 μM concentrations. Guided by a kinetic study (see Additional file 1), the 1 h treatment period was found to be optimal for all concentrations tested for the detection of caspase-8 initiation. To authenticate the caspase-8 involvement, cells were pre-incubated with 10 μM and 30 μM of caspase-8 inhibitor, z-IETD-fmk (Z-Ile-Glu-Thr-Asp-fmk) for 30 min and then exposed to each tocotrienol isomer. Caspase-8 colorimetric assay (GenScript, USA) was employed to quantify the caspase-8 activity in treated cell lines according to manufacturer’s instructions. Treated cells (in the absence or presence of caspase-8 inhibitor) were first collected into microcentrifuge tubes and centrifuged at 2,200 rpm for 5 min. Following two washes of pelleted cells with PBS, ice-cold lysis buffer (50 μl) containing 0.5 μl dithiothreitol (DTT) and 0.25 μl phenylmethanesulfonylfluoride (PMSF) was added into the collected cells and mixed well. The cells were then incubated on ice for 45 min with intermittent vortexing for 4 times, each for 10 sec. The cells were then subjected to centrifugation at 10,000 rpm for 1 min. Supernatants (50 μl) were transferred into wells of a 96-well plate; 50 μl of 2 x reaction buffer (containing 0.5 μl DTT and 0.25 μl PMSF) and 5 μl of caspase-8 substrate (IETD-*p*NA) were added to each sample. Samples were then incubated at 37°C for 1 h in the dark. The cleavage of labeled substrate IETD-*p*NA into chromophore *p*-nitroanilide (*p*NA) was determined by measuring the absorbance (optical density, OD) at 405 nm using a 96-well microplate reader (Thermo Fisher Scientific, USA). The result of the induced caspase-8 activity was obtained by computing OD_inducer_/OD_negative controls_ with the background OD values from cell lysates and buffers subtracted.

### Determination of Bid and cytochrome *c*activities by ELISA

The detection of human Bcl-2 homology domain 3 or BH-3 interacting domain death agonist (Bid) and cytochrome *c* was conducted by using enzyme-linked immunosorbent assay (ELISA). A total of 1 × 10^6^ cells/ml were seeded and grown in 60 mm^2^ petri dish. The cells were then treated with IC_50_ and IC_max_ concentrations of tocotrienol isomers (Table 1) for 4 h, alongside with an untreated negative control included. In order to study the correlation of caspase-8 with Bid and cytochrome *c* in the whole apoptotic execution, the cells were pre-incubated with 50 μM of caspase-8 inhibitor, z-IETD-fmk for 30 min prior tocotrienol treatment. The treated cells were diluted with 1 × PBS to reach cell density at 1 × 10^8^/ml and then stored overnight at −20°C. Cell lysates were centrifuged at 5,000 × *g* for 5 min at 4°C. The supernatant was collected and tested for standard ELISA procedures on Bid and cytochrome *c* levels according to manufacturer’s protocol (CUSABIO, USA).

### Western blotting assay for Bax detection

A total of 1 × 10^6^ cells/ml were seeded and grown in 100 mm^2^ petri dish. The cells were then treated with the IC_50_ concentration of each tocotrienol isomer (Table 1) for 24 h. Total protein (100 μg per well) was loaded onto a 10% SDS-PAGE followed by standard electrophoresis and electro-blotting transfer procedures. The blotted membrane was blocked with 10% milk for 24 h at 4°C. Membrane was rinsed thrice with Tris Buffer Saline Tween (TBST) (8 g/L Na Cl; 2.42 g/L Tris and 0.05% Tween 20; pH 7.6), each for 10 min. The membrane was incubated with the primary rabbit monoclonal antibody against Bax (Cell Signaling Technology, USA) (1: 1,000 diluted in TBST containing 5% milk) for 24 h at 4°C. Meanwhile, detection of beta-actin was used as a protein loading control. Following 3-time TBST washes (each for 10 min), the membrane was then incubated with secondary anti-rabbit antibody (1: 4,000 diluted in TBST) (Cell Signaling Technology, USA) for 1 h at room temperature with gentle shaking. After similar washing steps, LumiPico ECL kit (ShineGene, China) was used to detect the immobilized protein on the membrane using chemiluminescent method according to the manufacturer’s instructions.

### Mitochondrial membrane permeability assessment assay

Mitochondrial membrane permeability (MMP) was measured using a mitochondrial apoptosis detection kit as described by the manufacturer (Genscript, USA). Briefly, about 1 × 10^6^ cells were seeded on chamber slides (Lab-Tek, USA) for 24 h. In the presence or absence of caspase-8 inhibitor z-IETD-fmk, cells were treated with IC_50_ and IC_max_ concentrations of alpha-, gamma- and delta-tocotrienols (Table 1) for 3 h and 24 h; and vinblastine was tested at 0.011 μM and 11 μM concentrations for comparison. Following a washing step with PBS, the cells were immersed in 1 ml of diluted JC-1 reagent and incubated for 20 min. More than 200 cells in each replicate were analyzed instantly under an epifluorescence microscope (Nikon, Japan) using a band-pass filter. Cells that appeared in red fluorescence indicated MitoCapture JC-1 reagent accumulation in the intact mitochondria of healthy cells whereas cells that with changed MMP would fluoresce in green.

### Statistical analysis

The concentration-response curves were plotted by using probit or logistic analysis and the IC_50_, IC_80_ and IC_max_ values were then calculated by using Graphpad Prism (version 5) bio-statistical software. In general, quantitative experimental data were expressed as mean ± standard deviation (SD) generated from triplicates performed in three separate occasions for each group. When necessary, a standard curve was created by using the same software for target quantitation purpose. Unpaired or independent *t* test and ANOVA (using completely randomized design, CRD) were used to compare between two groups or to compare between groups. The level of statistical significance was set at *p* < 0.05.

## Results

### Cytotoxic effects of cancer cells treated with tocotrienols

**Figure 1:**
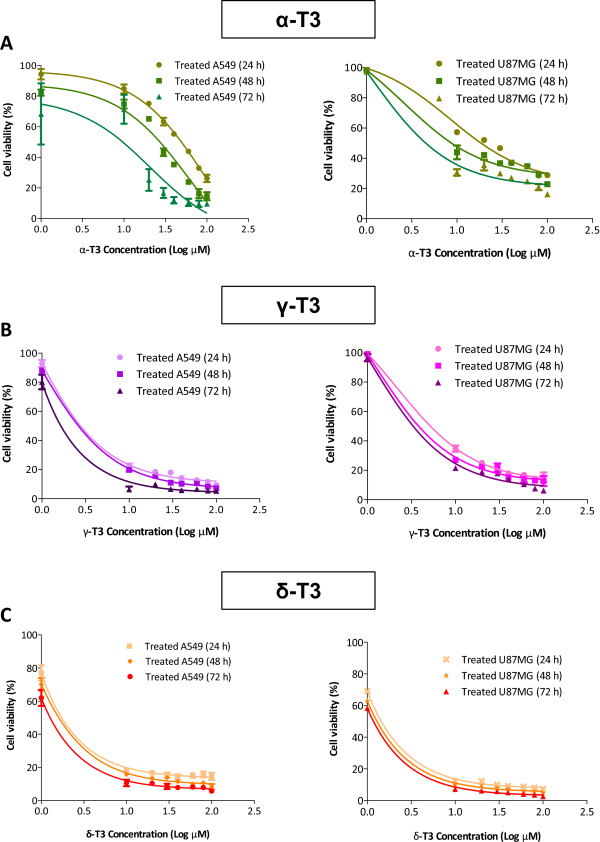
***Effects of different concentrations and treatment periods of tocotrienols on cell viability of A549 and U87MG cells.*** **(A)** Alpha-, **(B)** gamma- and **(C)** delta-tocotrienols show significant cytotoxic effects (*p* < 0.05) against both cancer cells in a concentration- and time-dependent manner. Cell viability is presented as percentage where negative vehicle control cells were regarded as 100% viable. Data are presented as mean ± SD (n = 9). α-T3: alpha-tocotrienol; γ-T3: gamma-tocotrienol; δ-T3: delta-tocotrienol.

The effects of various concentrations of alpha-, gamma- and delta-tocotrienols on the proliferation of both A549 and U87MG cell lines after defined treatment periods are shown in Figure 1. Treatments with alpha-, gamma- and delta-tocotrienols at the range between 1 μM and 100 μM significantly inhibited (*p* < 0.05) the cell viability in a concentration- and time-dependent manner. The IC_50_ values for alpha-, gamma- and delta-tocotrienols at 24, 48 and 72 h are shown in Table 2. Increased concentrations in the treatment had resulted in reduced cell numbers and enhanced cell death. On the other hand, all tocotrienol isomers were found not causing apparent impairment towards the non-cancerous MRC5 cells where IC_50_ > 100 μM. Overall results showed that delta-tocotrienol exhibited the most potent cytotoxicity towards both A549 and U87MG cells, followed by gamma-tocotrienol and then alpha-tocotrienol. Delta-tocotrienol seemed to possess comparable antiproliferative activity towards both A549 and U87MG cells, whereas, gamma-tocotrienol possessed 1.5 times lower IC_50_ value in A549 than that of U87MG cells. As opposed to that, alpha-tocotrienols was found to possess 3 times lower IC_50_ value in U87MG when compared to A549 cells. In comparison to tocotrienols, vinblastine possessed higher cytotoxic potency with lower IC_50_ values for both cancer cell lines, i.e. < 0.055 μM (0.01 μg/ml for A549 and 0.05 μg/ml for U87MG cells, respectively as published previously [21]).

**Table 2:** **Mean IC**_**50**_ **values of A549 and U87MG cells receiving treatments of tocotrienols**

	Mean IC _50_ Concentration (μM)					
Cell lines	A549			U87MG		
Treatment period	24 h	48 h	72 h	24 h	48 h	72 h
Alpha-tocotrienol	5.46 ± 0.8	4.93 ± 0.4	4.61 ± 1.7	2.57 ± 0.1	1.84 ± 0.2	1.53 ± 0.4
Gamma-tocotrienol	2.84 ± 0.1	2.56 ± 0.2	1.72 ± 0.2	5.02 ± 0.1	3.75 ± 0.1	3.32 ± 0.3
Delta-tocotrienol	1.71 ± 0.4	1.60 ± 0.1	1.27 ± 0.2	1.45 ± 0.1	1.29 ± 0.1	1.13 ± 0.9

Each value is expressed as a mean ± SD (n = 9).

### Morphological alterations of cancer cells treated with tocotrienols

**Figure 2:**
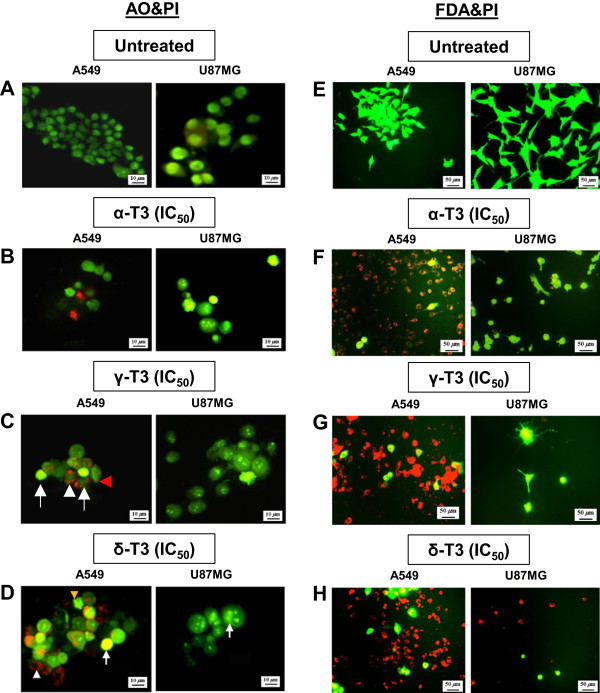
***Representative AO&PI and FDA&PI staining images of A549 and U87MG cells treated with tocotrienols.*** Following a treatment period of 24 h, **(A,E)** untreated vehicle control cells show normal appearance with no prominent apoptotic and necrotic cells. In AO&PI staining, A549 and U87MG cells treated with IC_50_ of **(B)** alpha-, **(C)** gamma- and **(D)** delta-tocotrienols show early apoptosis exhibiting a bright-green nucleus as condensation of chromatin (white arrows), and late apoptosis demonstrating cellular blebbing and nuclear margination (white and red arrowhead). FDA&PI stained A549 and U87MG cells treated with IC_50_ of **(F)** alpha-, **(G)** gamma- and **(H)** delta-tocotrienols show cell death with higher red fluorescence evidenced as compared to the untreated cells **(E)**. Bar (AO&PI): 10 μm; Bar (FDA&PI): 50 μm; α-T3: alpha-tocotrienol; γ-T3: gamma-tocotrienol; δ-T3: delta-tocotrienol.

Following histochemical and fluorescence staining, morphological characteristics of cellular apoptotic death such as nuclear fragmentation, chromatin condensation, multinucleated cells, vesicles formation, cytoplasmic extension, cell blebbing and formation of apoptotic bodies were evident microscopically in all alpha-, gamma- and delta-tocotrienols treated A549 and U87MG cancer cells at their respective IC_50_ and IC_80_ concentrations. Treated MRC5 cells exhibited a mild degree of cellular stress morphologies such as tiny vesicles formation and cytoplasmic extension. A few representative images are shown in Figure 2. Vehicle control untreated cancer cells were observed with a green intact nuclear structure (Figure 2A). Early apoptosis was detected by the evidence of obvious intercalated AO within the fragmented DNA. Nuclear chromatin condensation was observed during mid-apoptosis (Figure 2B-D). Meanwhile, the later stages of apoptosis can be evident by changes such as the presence of reddish-orange colour in cells, owing to the binding of AO to denatured DNA and penetration of PI stain through the nuclear membranes that losing their permeability (Figure 2B-D).

Differential scoring of treated cells stained with AO&PI for the quantification of viable, early apoptotic, late apoptotic and secondary necrotic morphologies showed that all alpha-, gamma- and delta-tocotrienols were found to trigger morphological features related to apoptosis in a concentration- and time-dependent manner. As referred to Table 3, alpha-tocotrienol was found to cause the highest percentage of early apoptosis in A549 cells, whereas in the case of U87MG cells, the highest percentage of cells was at the mid stage of apoptosis. Gamma-tocotrienol caused the highest percentage of mid stage apoptotic cells, whereas, delta-tocotrienol produced the highest late stage apoptotic cells in both A549 and U87MG cells.

**Table 3:** **Percentage of tocotrienols-treated A549 and U87MG cells categorized at different stages of apoptosis**

Treatment at IC _50_	Cells categorized at different phases of apoptosis (%)					
	A549			U87MG		
	Early	Mid	Late	Early	Mid	Late
Alpha-tocotrienol	57.6	28.5	15.1	22.3	70.7	8.8
Gamma-tocotrienol	11.4	63.1	26.3	35.4	48.2	17.1
Delta-tocotrienol	6.0	36.4	58.9	4.3	23.8	73.1

Values are calculated based on 200 cells.

Overall results indicated that all alpha-, gamma- and delta-tocotrienols showed impressively significant (*p* < 0.05) apoptotic effects in both A549 and U87MG cells as compared to the vehicle control untreated cells. There was no statistically significant (*p* > 0.05) difference in necrotic counts at different treatment times. Some MRC5 cells were found undergoing apoptosis when treated with 50 μM of alpha-, gamma- and delta-tocotrienols, however, the number of apoptotic cells was < 20% and statistically insignificant (*p* > 0.05).

Under FDA&PI fluorescent staining (Figure 2E-H), the numbers of survived cells with changed morphology and dead cells which stained in red were highly dependent on the concentrations of alpha-, gamma- and delta-tocotrienols tested. Maximal quantities of cell death found in A549 and U87MG cells, respectively, receiving the treatment of IC_80_ concentrations were 77% and 95% for alpha-tocotrienol, 97% and 84% for gamma-tocotrienol, and 100% in both cells for delta-tocotrienol. The frequency of dead cells in the vehicle control untreated A549 and U87MG cells did not exceed 1% at the end of the experiment and necrotic cells were not observed.

### DNA damage pattern of cancer cells treated with tocotrienols

**Figure 3:**
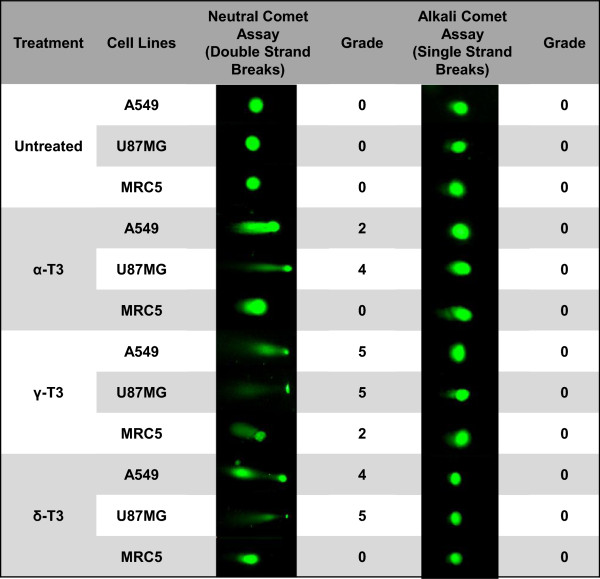
***Representative neutral and alkaline comet profiles of A549, U87MG and MRC5 cells treated with tocotrienols*** **.** Based on the comet profiles, mean DNA damage were scored for A549, U87MG and MRC5 cells in response to alpha-, gamma- and delta-tocotrienols treatments for 72 h. Untreated cells show intact nuclei without comet-like appearance. Cells treated with IC_50_ values of alpha-, gamma- and delta-tocotrienols showing U87MG cells have higher scores of DSBs than A549 cells and MRC5 DNA damage is negligible. No SSBs are found in all treated cells. α-T3: alpha-tocotrienol; γ-T3: gamma-tocotrienol; δ-T3: delta-tocotrienol.

The induction of apoptotic cell death by alpha-, gamma- and delta-tocotrienols was examined for the involvement of DNA damage in A549 and U87MG cells by comet assay. Alpha-, gamma- and delta-tocotrienols treated A549 and U87MG cells showed well-formed comets while the vehicle control untreated cancer and normal cells did not demonstrate any comet-like appearance (Figure 3) indicating no DNA damage or breakage. All tocotrienol isomers were found to produce only double strand breaks (DSBs, neutral comets) but no single strand breaks (SSBs, alkali comets) in both A549 and U87MG cells (Figure 3). For instance, the induction of apoptosis in both cancer cells was confirmed. It could be seen that gamma-tocotrienol did have certain DNA damaging effects on the non-cancerous MRC5 cells, causing a small amount of DSBs but the DNA damage score was trivial as compared to that observed in treated A549 and U87MG cells (Figure 3). The DNA damage scores for both gamma- and delta-tocotrienols were comparable, while alpha-tocotrienol was observed to cause less severe DNA damage than the former two isomers. Similarly, vinblastine also caused DSBs only by which its comet results can be referred to our previously published report [21].

### Cell cycle phase distribution of cancer cells treated with tocotrienols

**Figure 4:**
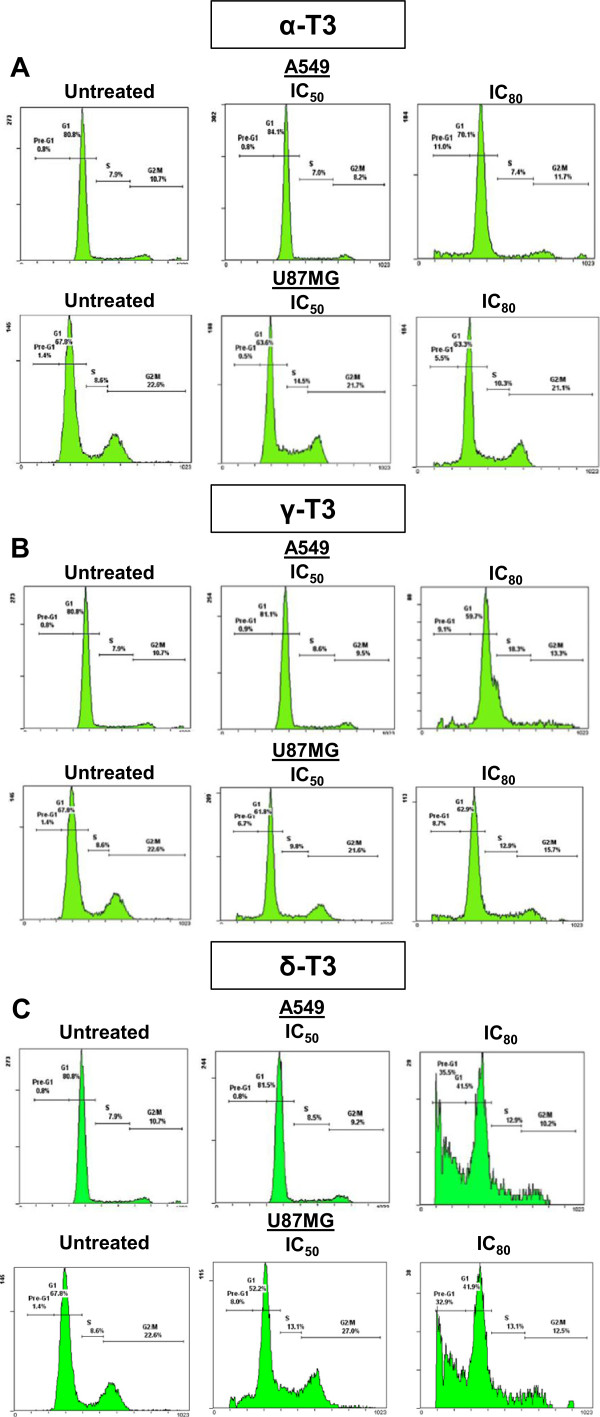
***Representative flow cytometric profiles of A549 and U87MG cells treated with tocotrienols.*** The effects of **(A)** alpha-, **(B)** gamma- and **(C)** delta-tocotrienols on cell populations of untreated and treated A549 and U87MG cells at IC_50_ and IC_80_ concentrations for 48 h are shown. The fact with increased cell populations at the Pre-G_1_ phase of both treated A549 and U87MG cells confirms DNA fragmentation and apoptosis without doubt. α-T3: alpha-tocotrienol; γ-T3: gamma-tocotrienol; δ-T3: delta-tocotrienol.

Treatments of A549 and U87MG cells with alpha-, gamma- and delta-tocotrienols increased the ratio of G_0_/G_1_ phase (refer to Pre-G_1_ staining) as compared to the control groups at both 24 h (data not shown) and 48 h as shown in Figure 4. Alpha-tocotrienol at IC_80_ concentration increased cell cycle distribution at G_0_/G_1_ stage from 0.8% (untreated) to 11.0% (treated) and 1.4% to 5.5% in A549 and U87MG cells, respectively (Figure 4A). Comparable trend was seen in gamma-tocotrienol-treated A549 and U87MG cells at G_0_/G_I_ stage with increment from 0.8% in the untreated to 9.1% and 1.4% to 8.7%, respectively (Figure 4B). Among all tocotrienol isomers tested, delta-tocotrienol at IC_80_ concentration was found to execute the highest percentage of cell distribution at G_0_/G_1_ stage with more than 34-fold increase in A549 cells and 31-fold increase in U87MG cells (Figure 4C). The significant increase of the cell proportion at G_0_ stage, and the decrease of the cell proportion at G_I_ stage for all alpha-, gamma- and delta-tocotrienols-treated A549 and U87MG cells were in fact dose-dependent (*p* < 0.05).

### Caspase-8, Bid and Bax activities of cancer cells treated with tocotrienols

**Figure 5:**
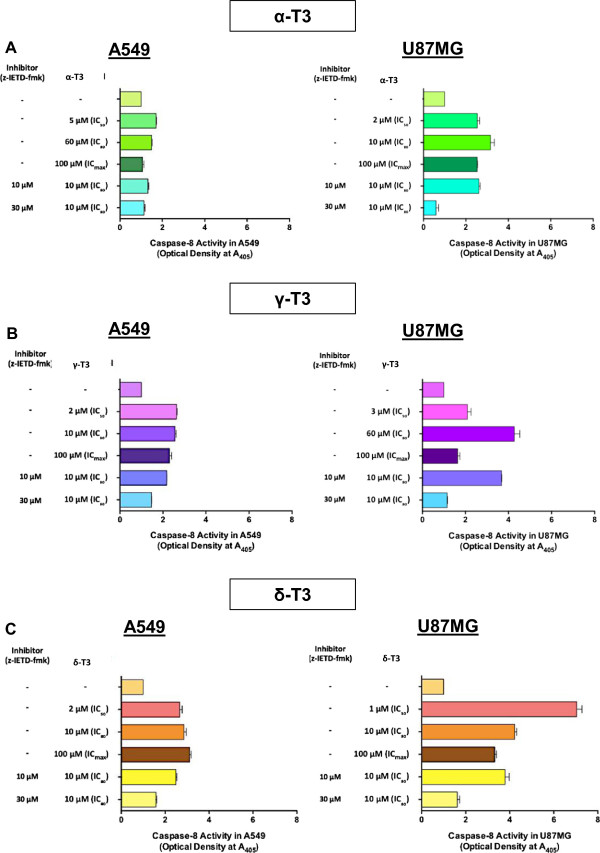
***Effects of tocotrienols on caspase-8 activities of A549 and U87MG cells.*** **(A)** Alpha-, **(B)** gamma- and **(C)** delta-tocotrienols show distinguished effects on caspase-8 activities of untreated and treated A549 and U87MG cells for 1 h treatment period at the concentrations of IC_50_, IC_80_ and IC_max_ with or without caspase-8 inhibitor, z-IETD-fmk (10 μM and 30 μM). Generally, alpha-, gamma- and delta-tocotrienols are able to trigger caspase-8 activities in both treated A549 and U87MG cells. All tocotrienol isomers induced approximately 2 times greater caspase-8 activation in U87MG cells as compared to A549 cells. α-T3: alpha-tocotrienol; γ-T3: gamma-tocotrienol; δ-T3: delta-tocotrienol.

Alpha-, gamma- and delta-tocotrienols were able to trigger caspase-8 activity in both A549 and U87MG cells comparing with the untreated groups, which remained at its basal level (Figure 5). Overall results indicated that all tocotrienol isomers induced approximately 2 times greater caspase-8 activation in U87MG cells as compared to A549 cells. The induction peaks of alpha- and gamma-tocotrienols were detected at its IC_50_ and IC_80_ concentrations in A549 and U87MG cells, respectively, whereas delta-tocotrienol induction peaks were detected at its IC_max_ concentration in A549 cells and IC_50_ concentration in U87MG cells. Study showed that vinblastine did not induce caspase-8 activation at all concentrations and treatment time points tested as the caspase-8 level was similar to that of untreated cells (data not shown). In the presence of caspase-8 inhibitor, z-IETD-fmk, a clear decrease in alpha-, gamma- and delta-tocotrienols-induced caspase-8 activities was observed somewhat linearly related to the z-IETD-fmk concentration used (Figure 5). The percentage of inhibitory effects of 30 μM z-IETD-fmk in all alpha-, gamma- and delta-tocotrienols-treated A549 and U87MG cells are presented in Table 4.

**Table 4:** **Percentage of inhibition of 30 μM z-IETD-fmk on caspase-8 activities of tocotrienols-treated A549 and U87MG cells**

Treatment at IC _80_	Inhibitory effects of 30 μM z-IETD-fmk (%)	
	A549	U87MG
Alpha-tocotrienol	26.7	100.0
Gamma-tocotrienol	41.2	72.5
Delta-tocotrienol	42.8	61.9

**Figure 6:**
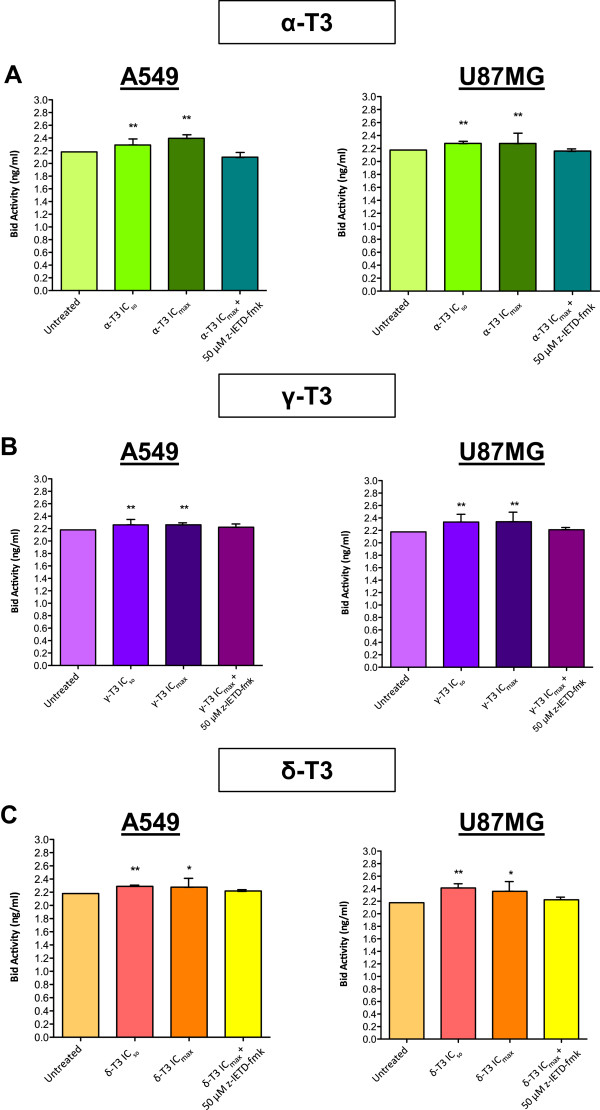
***Effects of tocotrienols on Bid protein activities of A549 and U87MG cells.*** **(A)** Alpha-, **(B)** gamma- and **(C)** delta-tocotrienols are able to induce higher Bid protein levels on treated A549 and U87MG cells at IC_50_ and IC_max_ concentrations for 4 h than the basal levels of untreated cells. However, 50 μM of caspase-8 inhibitor, z-IETD-fmk is able to suppress the augmentation effects of tocotrienols on Bid protein activities. *Significance levels (*p < 0.05; **p < 0.01; ***p < 0.001) are set to compare between samples. α-T3: alpha-tocotrienol; γ-T3: gamma-tocotrienol; δ-T3: delta-tocotrienol.

Figure 6 illustrates that the levels of Bid protein in A549 and U87MG cancer cells treated with alpha-, gamma- and delta-tocotrienols increased significantly as compared to the basal level of untreated samples (*p* < 0.05). However, when caspase-8 inhibitor, z-IETD-fmk was added to individual samples prior treatment of the tocotrienols, it could be seen that the level of Bid protein was prohibited (Figure 6).

**Figure 7:**
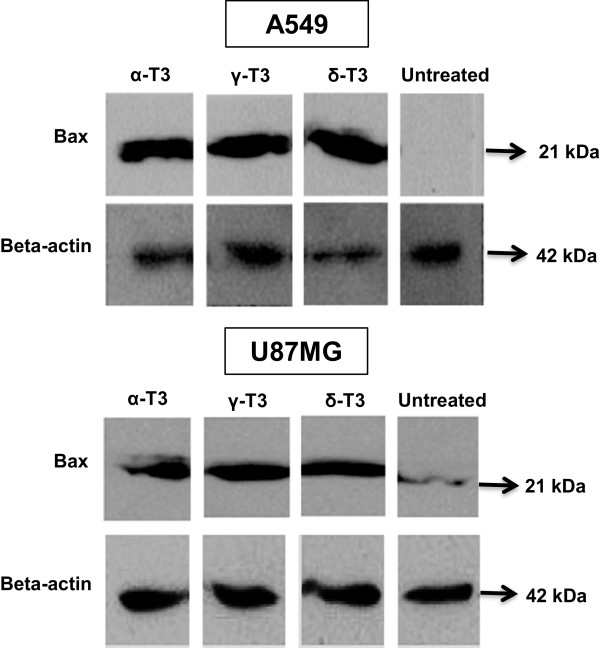
***Western profiles of Bax protein expression in A549 and U87MG cells treated with tocotrienols.*** Following 4 h treatment period, Bax protein expression at a molecular size of 21 kDa reacted with anti-Bax antibody is detected in A549 and U87MG cells treated with alpha-, gamma- and delta-tocotrienols by which its density is higher than that of untreated counterparts. Beta-actin is served as a protein loading control. α-T3: alpha-tocotrienol; γ-T3: gamma-tocotrienol; δ-T3: delta-tocotrienol.

On the other hand, Figure 7 shows that Bax protein was detected as a specific protein band at 21 kDa in alpha-, gamma- and delta-tocotrienols-treated A549 and U87MG cancer cells with higher expression level. In contrast, there were no band and only a faint band obtained for untreated A549 and U87MG cells, respectively. Results had been validated with a protein loading control, β-actin with a molecular size of 42 kDa that was detected in all protein samples.

### Alterations of mitochondrial permeability of cancer cells treated with tocotrienols

**Figure 8:**
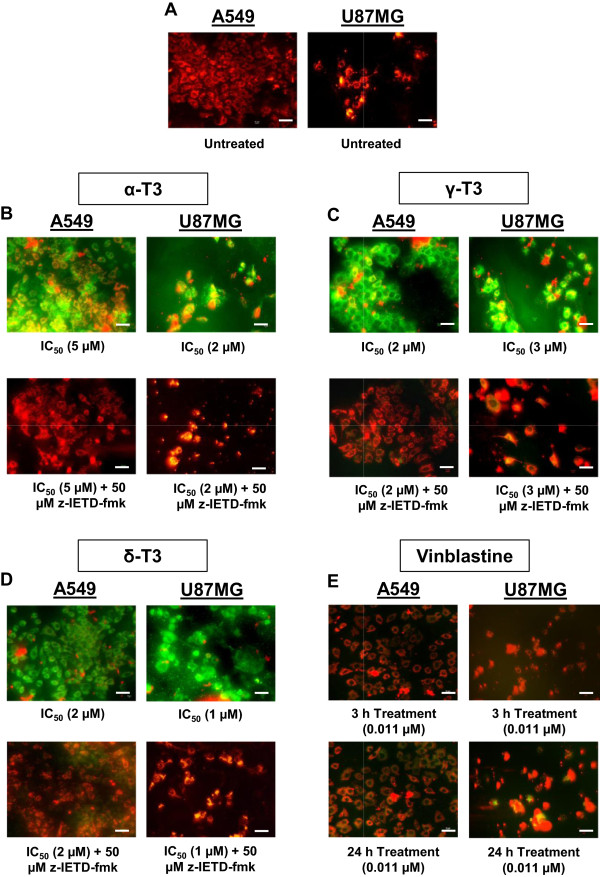
***Mitochondrial membrane permeability (MMP) alterations in A549 and U87MG cells treated with tocotrienols and vinblastine.*** Representative images showing the effects of **(B)** alpha-, **(C)** gamma- **(D)** delta-tocotrienols at 3 h treatment period on MMP of untreated **(A)** and treated A549 and U87MG cells in the presence or absence of caspase-8 inhibitor z-IETD-fmk. Untreated cancer cells possess intact mitochondrial membranes exhibiting intense red fluorescence, whereas treated cells show deep green fluorescence owing to loss of mitochondrial permeability. Cells pre-treated with z-IETD-fmk are not affected by either tocotrienol treatment, which still exhibit intense red fluorescence. **(E)** Representative images showing the effects of vinblastine at 3 h and 24 h treatment periods on MMP of treated A549 and U87MG cells. Vinblastine induces MMP very minimally where majority of treated A549 and U87MG cells show strong red fluorescence without being affected by the increased treatment period and concentration used. Bar: 20 μm. α-T3: alpha-tocotrienol; γ-T3: gamma-tocotrienol; δ-T3: delta-tocotrienol.

The effects of alpha-, gamma- and delta-tocotrienols on the mitochondrial membrane permeability (MMP) of A549 and U87MG cells were evaluated for potential involvement of the intrinsic apoptotic signalling pathway. As shown in Figure 8A, JC-1 reagent taken up in the mitochondria of vehicle control untreated A549 and U87MG cells exhibited intense red fluorescence indicating the cells were healthy with intact mitochondria. However, all cancer cells treated with alpha-, gamma- and delta-tocotrienols showed intense green fluorescence demonstrating JC-1was not able to accumulate in the mitochondria, which indicates the loss of MMP (Figure 8B-D). The intensity of the green fluorescence signal increased in a concentration-dependent manner for all tocotrienols-treated cancer cells but was comparable for both 3 h and 24 h treatment periods. Captivatingly, cells that pre-treated with z-IETD-fmk were not affected by the treatments of alpha-, gamma- and delta-tocotrienols where JC-1 accumulation within mitochondria was still apparent (Figure 8B-D). The apoptotic event had been inhibited by z-IETD-fmk and it is suggested that caspase-8 has a direct effect on MMP in this case.

On the contrary, vinblastine was found to disrupt the MMP of both cancer cells negligibly where only very minimum amount of green fluorescence could be seen (Figure 8E) and the signal was much weaker than that of triggered by any tocotrienol isomer tested. This indicates the cancer cells treated by vinblastine retained intact mitochondrial membranes. This phenomenon persisted even when the treatment period was increased to 24 h (Figure 8E) and higher concentration at 11 μM was used, majority of the cells were still fluoresced red.

### Cytochrome *c*release of cancer cells treated with tocotrienols

**Figure 9:**
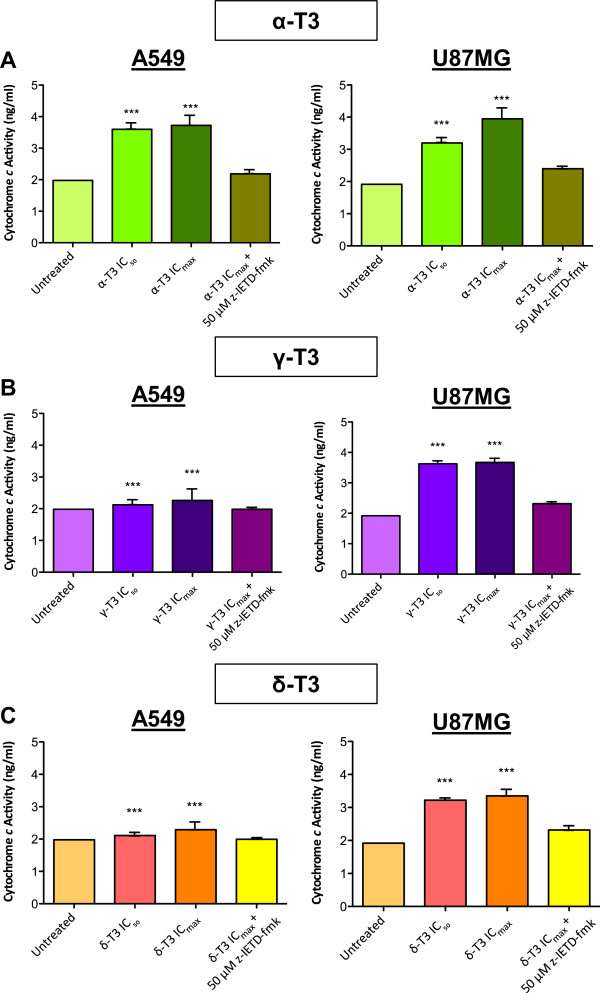
***Effects of tocotrienols on cytochrome c release of A549 and U87MG cells.*** Cytochrome *c* levels of A549 and U87MG cells treated with **(A)** alpha-, **(B)** gamma- and **(C)** delta-tocotrienols at IC_50_ and IC_max_ concentrations for 4 h with or without pretreatment of 50 μM of caspase-8 inhibitor, z-IETD-fmk are detected by ELISA. Significantly higher level of cytochrome *c* is released from all alpha-, gamma- and delta-tocotrienols-treated A549 and U87MG cells (*p* < 0.05) than that of untreated counterparts. The caspase-8 inhibitor, z-IETD-fmk has a direct effect in suppressing the cytochrome *c* release from treated cells. *Significance levels (*p < 0.05; **p < 0.01; ***p < 0.001) are set to compare between samples. α-T3: alpha-tocotrienol; γ-T3: gamma-tocotrienol; δ-T3: delta-tocotrienol.

When compared to untreated groups, the cytochrome *c* was released at a higher level from both A549 and U87MG cells treated with alpha-, gamma- and delta-tocotrienols (*p* < 0.05). The cytochrome *c* level in alpha-, gamma- and delta-tocotrienols-treated cells came close to the basal level of the untreated vehicle control cells when caspase-8 inhibitor, z-IETD-fmk was added prior to the treatment (Figure 9). Upon treatment with IC_50_ and IC_max_ concentrations of alpha-, gamma- and delta-tocotrienols, it could be seen that the cytochrome *c* levels increased intensively. The increment percentage is captured in Table 5. Overall results showed that alpha-, gamma- and delta-tocotrienols induced higher level of cytochrome *c* release in U87MG than A549 cells.

**Table 5:** **Increment percentage of cytochrome** ***c*** **release in A549 and U87MG cells treated with tocotrienols**

	Increment of Cytochrome ***c*** Release (%)			
Treatment	A549		U87MG	
	IC _50_	IC _max_	IC _50_	IC _max_
Alpha-tocotrienol	82.2	88.8	65.6	104.7
Gamma-tocotrienol	7.6	14.5	89.1	91.1
Delta-tocotrienol	10.2	19.8	68.4	78.9

Values are calculated as a comparison to the untreated groups.

## Discussion

Numerous previous studies had demonstrated that tocotrienols, particularly the most widely studied gamma isoform to possess anticancer properties in addition to inflammatory and antioxidant activities [6, 11–19]. In fact, evidences have revealed that each vitamin E isomer has its own pharmacodynamic profile [24, 25]. The precise reason why certain isomers are more effective than the others as antitumor agents is still unclear; but undoubtedly, the study on anticancer effects of individual isomers in different cell types is necessary to obtain more conclusive answers. Although all tocotrienol isomers share the same basic chemical structure characterized by a long phytyl chain attached at the 1-position of a chomane ring, it was found that the location of the methyl groups at the head region are critical in determining the antiproliferative and apoptotic activities of individual isoforms [26–28]. Both delta- and gamma-tocotrienols are categorized as desmethyl tocotrienol owing to their fewer amounts of methyl groups and have been proven to confer higher potency than the other isoforms [27, 28]. Hence, it is of our particular interest to investigate whether alpha- and delta-tocotrienol would possess similar cytotoxic capability as with what reported by other studies for gamma-tocotrienol, or would they be possibly better. In fact, studies done on delta- and alpha-tocotrienols are relatively limited as compared to gamma-tocotrienol; additionally, effects of tocotrienols in human glioblastoma (U87MG) are very rare if not none in any investigations which are worthwhile to report. Thus, the anticancer properties of alpha-, gamma- and delta-tocotrienols in both A549 and U87MG cells are described herein.

Current findings showed that treatments with alpha-, gamma- and delta-tocotrienols significantly inhibited the growth of both A549 and U87MG cells in a concentration- and time-dependent manner (*p* < 0.05) but caused no apparent harm towards non-cancerous MRC5 cells. The IC_50_ values obtained especially for gamma- and delta-tocotrienols (Table 2) fulfilled the required anticancer potency as set by National Cancer Institute (NCI), i.e. < 4 μM [29, 30] which warrant further investigations on their anticancer mechanisms. Delta-tocotrienol with the lowest IC_50_ values was found to exhibit the highest antiproliferative potency against both A549 and U87MG cells (Table 2) as compared to the other isomers. Interestingly, alpha-tocotrienol with a weak antiproliferative effect on A549 cells, was found to possess a slightly higher potency in inhibiting growth of U87MG cells than gamma-tocotrienol. This suggests that alpha-tocotrienol might possess selectivity potential towards different cancer types, i.e. U87MG cells in this case.

Morphological characteristics of apoptosis have been revealed in alpha-, gamma- and delta-tocotrienols-treated A549 and U87MG cells via histochemical and fluorescence staining techniques. These include cytoplasm vacuolization, cell shrinkage, cell blebbing, formation of stretched nuclei, chromatin condensation at the nuclear periphery, appearances of apoptotic nuclei and multinucleated cells. In all cases, the number of cells with changed morphology was dependent on the treatment concentrations of tocotrienols. Quantification of apoptosis with differential scoring of treated and untreated cancer cells revealed a significant (*p* < 0.05) increase in the number of apoptotic cells over time but with insignificant difference between numbers of necrotic cells in these two cells. All these observations signified that alpha-, gamma- and delta-tocotrienols triggered apoptosis instead of necrotic effects.

The induction of apoptosis in alpha-, gamma- and delta-tocotrienols-treated cancer cells had been further confirmed by comet assay (Figure 3). Majority of DNA migrated towards the tail of the comet after treatment, which is distinguished to the phenomenon of necrosis, where the majority of damage would remain in the head of the comet, although some smaller fragments could escape [31–33]. Conjoining with morphological findings where chromatin condensation and fragmentation, apoptotic bodies and so on were evident, it could be concluded that cellular DNA damage in all tocotrienols-treated cells was definitely caused by apoptosis. Generally, chemicals or ionizing radiation may result in many types of DNA damage including SSBs, DSBs, base damage, and DNA-DNA or DNA-protein crosslinks [34, 35]. Current comet profiles showed only DNA DSBs (neutral comets) were induced by alpha-, gamma- and delta-tocotrienols in both treated A549 and U87MG cancer cells. In comparison with previously published results, vinblastine also generated only DSBs without SSBs seen in A549 and U87MG cancer cells [20, 21] but with lower DNA damage scores than those comets induced by these three tocotrienols (Figure 3). Vinblastine has been known to trigger apoptosis through inhibiting the formation of microtubules and causes microtubules assemblage [36]. Our current results therefore suggest that alpha-, gamma- and delta-tocotrienols, which induced similar DSBs, might also act on microtubules in both A549 and U87MG cells.

Flow cytometric profiles of both A549 and U87MG cells treated with alpha-, gamma- and delta-tocotrienols (Figure 4) revealed an upsurge cell population at the Pre-G_1_ phase, which have further confirmed the occurrence of DNA fragmentation and apoptosis without doubt. Considering the fact that p21 and p53 are the most important components controlling the arrest in the G_1_ and the G_2_ checkpoints [37] but not at the G_0_ and S phase checkpoints, the insignificant (*p* > 0.05) cell cycle arrest results in the G_1_ and G_2_ checkpoints suggest that p21 and p53 are most probably not involved in alpha-, gamma- and delta-tocotrienols triggered apoptosis. As mentioned above that the stabilization and activation of the p53 pathway is usually triggered by the presence of DNA damage that cannot be repaired [35, 38]; thus guided by the fact that apoptosis initiated by alpha-, gamma- and delta-tocotrienols did not cause any SSBs, this again rules out the possibility for the involvement of p53 pathway [37–39]. However, further investigation shall be implemented to support such speculations.

Caspase-mediated apoptosis in most cells is known to involve the activation of either death receptor (extrinsic) pathway or the mitochondrial (intrinsic) pathway and sometimes, both pathways simultaneously. Current findings showed that the caspase-8 activities in A549 and U87MG cells were significantly enhanced after receiving the treatments of all alpha-, gamma- and delta-tocotrienols (Figure 5). The activation of caspase-8 has been verified by the use of caspase-8 inhibitor, z-IETD-fmk that could significantly reduce the elevated level or even revert back to its basal state (Figure 5). A good association between caspase-8 activation and downstream activities was further demonstrated on Bid (Figure 6), Bax (Figure 7), MMP (Figure 8) and cytochrome *c* (Figure 9) assessments in alpha-, gamma- and delta-tocotrienols-treated A549 and U87MG cells. When a similar caspase-8 inhibitor, z-IETD-fmk was pre-added to the cells, each respective level of the abovementioned activities had substantially been suppressed. These observations support the hypothesis that extrinsic and intrinsic apoptotic pathways are interconnected in alpha-, gamma- and delta-tocotrienols-initiated apoptosis in both A549 and U87MG cells.

Generally, the initiation of the death receptor pathway (or the extrinsic pathway) involves the recruitment of adaptor protein procaspase-8 by Fas-associated death domain (FADD) on the cell membrane to form the death-inducing signaling complex [40]. The formation of such signaling complex will subsequently lead to the cleavage and activation of procaspase-8 to caspase-8. Then, active caspase-8 can either stimulate caspase-3 directly resulting in an immediate apoptosis which is usually regulated by an abundance of inhibitors for apoptosis; or, it could sometimes require a more complicated but efficient mechanism, which involves the cross-talk between the extrinsic and the intrinsic pathways [40–42]. For instance, activated caspase-8 from the extrinsic pathway cleaves Bid (a BH-3 only pro-apoptotic member of the Bcl-2 family) into its truncated form [42, 43], which will then translocate into the mitochondria (intrinsic pathway) through the activation of other pro-apoptotic Bcl-2 family members, Bax and Bak [44, 45]. As a result, loss of mitochondrial membrane permeability occurred leading to the release of cytochrome *c*, followed by the interaction of CARD (caspase activation and recruitment domain) adaptor protein, APAF-1 (apoptotic protease activating factor 1) with procaspase-9, which eventually assemble into an apoptosome in the cytosol. This caspase-9 activation ultimately results in the cleavage and initiation of downstream effector caspases such as caspase-3 [45–47]. Ultimately, all alpha-, gamma- and delta-tocotrienols-treated A549 and U87MG cells are subjected to apoptotic cell death as observed in this study.

In fact, distinguished recent studies have suggested that different tocotrienol isomers might exhibit a different cellular mechanism of apoptosis in different cancer types. However, the specific apoptotic mechanisms induced by alpha-, gamma- and delta-tocotrienols in both brain and lung cancers are still unclear till date. A number of previous studies had revealed that gamma-tocotrienol actually acts as an inhibitor of nuclear factor-κB (NF-κB) p65 protein expression and nuclear translocation in the human colon cancer HT-29 cells, resulting in a direct effect on cell cycle progression and activation of the pro-apoptotic pathway [7]. Nevertheless, gamma-tocotrienol was also found in several other studies to possess cytotoxicity in human gastric carcinoma [15] and liver cancer cells [14] through the expression of Bcl-2 family proteins, increasing the release of cytochrome *c*, leading to the activation of procaspase-9 and caspase-3. These incidences further lead to the fragmentation of poly (ADP-ribose) polymerase (PARP), which is responsible in maintaining genomic stability and DNA-damage-triggered signaling cascade [14, 15]. In fact, a cleaved PARP by caspase-3 during apoptosis is an irreversible event, where at this stage a cell is fated to undergo apoptosis and is no longer able to respond to any DNA damage reparation [14, 15], this is widely recognized as a hallmark for cellular apoptosis [47].

As opposed to that, the involvement of caspase activation and Bcl-2 modulation was found not to be involved in gamma-tocotrienol-induced apoptosis in human breast cancer cells as discovered by Takahashi and Loo [6]. In their study, apoptosis initiated by gamma-tocotrienol in human breast cancer cells involved only the mitochondria-mediated death pathway. Meanwhile, gamma-tocotrienol was found in prostate cancer model to suppress cancer metastasis through the induction of mesenchymal-epithelial transition [16]. Nevertheless, there are some contradicting opinions that question the involvement of caspases and modulation of Bax/Bcl-2 in the gamma-tocotrienol executed apoptotic pathways [6, 7, 14, 15]. Recent studies of delta-tocotrienol on non-small cell lung cancer discovered that the anticancer activity induced by delta-tocotrienol is associated with its ability to decrease Notch-1, Hes-1, Survivin, MMP-9, VEGF, and Bcl-XL expressions, with additional decrease of binding activity in NF-κB-DNA [48, 49]. Our current study has complemented some of these findings where tocotrienols especially the delta isoform could induce a more effective apoptotic cell death in A549 and U87MG cancer cells via caspase-8-mediated Bid and Bax activations. Delta-tocotrienol is said to be a potent anticancer compound that can prevent tumor progression not only in non-small cell lung cancer [48, 49] but also in another type of cancer, i.e. glioma as demonstrated for the first time in our study. Based on the abovementioned contexts, it is suggested that tocotrienol isomers might inhibit the proliferation and invasion of cancers utilizing multiple molecular pathways, which are specific to cancer phenotypes.

## Conclusions

This study has provided a structural backbone in mapping the apoptotic mechanisms in all alpha-, gamma- and delta-tocotrienols induced apoptosis in both A549 and U87MG cells (see Additional file 2). It is now revealed that alpha-, gamma- and delta-tocotrienols induce apoptosis through the cross-talk of both the extrinsic and the intrinsic pathways. This process involves the induction of DSBs leading to the activation of caspase-8, resulting in the cleavage of Bid and activation of Bax, which Bax will further translocate into the mitochondrial membrane leading to the loss of MMP and causes the release of cytochrome *c*. This in turn will result in the execution of apoptosis in both A549 and U87MG cells. However, more studies are necessitated to provide further evidences especially for the very rarely studied glioblastoma. Future investigations could recruit other Bcl-2 family members, elucidate the involvement of the death receptor (Fas-L, Apo2L/Apo3L, or tumour necrosis factor-related apoptosis inducing ligand), association of p53, microtubule assemblage potential, participations of PARP, APAF-1 and downstream effector caspases to make a better conclusion for the apoptotic mechanisms triggered by tocotrienols. Nevertheless, findings obtained from this study have adequately suggested that delta-tocotrienol is indeed the most effective (higher induction rate) and efficient (shorter induction time) apoptotic executor among all isomers tested. Interestingly, tocotrienols have been reported to be capable of crossing the blood-brain barrier [50]. Therefore, it is ideal to use the most potent delta-tocotrienol for chemoprevention and chemotherapy in glioblastoma owing to its bioavailability in the brain.

## Electronic supplementary material

Additional file 1: **Caspase-8 kinetic study. Kinetic study of caspase-8 activity in delta-tocotrienol treated A549 cells.** In order to acquire the most optimum treatment time point for determining the caspase-8 initiation, a kinetic study was carried out in A549 cells receiving delta-tocotrienol at MIC (1 μM), IC_50_ (2 μM), IC_80_ (10 μM) and IC_max_ (100 μM) concentrations for different time intervals, i.e. 0 h, 0.5 h, 1 h, 2 h and 3 h. The 1 h incubation period was chosen for subsequent evaluation of cellular caspase-8 activity. MIC: minimum inhibitory concentration. (PPTX 115 KB)

Additional file 2: **Proposed apoptotic pathway.** Proposed apoptotic pathway induced by alpha-, gamma- and delta-tocotrienols. Tocotrienol isomers are proposed to induce apoptosis in A549 and U87MG cell lines with the involvement of cross-talk between extrinsic and intrinsic pathways based on the evidences gathered from caspase-8-dependent cleavage of Bid which led to Bax activation, mitochondrial membrane potential loss and eventually cytochrome *c* release, hence resulting the initiation of downstream effector caspases. (PPTX 68 KB)

## Abbreviations

DSBs: Double strand DNA breaks
SSBs: Single strand DNA breaks
α-T3: Alpha-tocotrienol
γ-T3: Gamma-tocotrienol
δ-T3: Delta-tocotrienol
AO&PI: Acridine orange and propidium iodide
H&E: Haematoxylin and eosin
FDA&PI: Fluorescein diacetate and propidium iodide
NCI: National Cancer Institute
ELISA: Enzyme-linked-immunosorbent assays
SCGE: Single cell gel electrophoresis
MMP: Mitochondrial membrane permeability
A549: Human lung adenocarcinoma
U87MG: Grade IV glioblastoma
MRC5: Normal lung fibroblast.
