# Supplementary material for: Cytotoxicity and apoptotic activities of alpha-, gamma- and delta-tocotrienol isomers on human cancer cells
Source: BMC Complement Altern Med. 2014 Dec 6;14:469. doi: 10.1186/1472-6882-14-469 (PMC4295404; doi:10.1186/1472-6882-14-469)
Supplement: Supplementary file 1 — Additional file 1: Caspase-8 kinetic study. Kinetic study of caspase-8 activity in delta-tocotrienol treated A549 cells. In order to acquire the most optimum treatment time point for determining the caspase-8 initiation, a kinetic study was carried out in A549 cells receiving delta-tocotrienol at MIC (1 μM), IC50 (2 μM), IC80 (10 μM) and ICmax (100 μM) concentrations for different time intervals, i.e. 0 h, 0.5 h, 1 h, 2 h and 3 h. The 1 h incubation period was chosen for subsequent evaluation of cellular caspase-8 activity. MIC: minimum inhibitory concentration. (PPTX 115 KB) [file 12906_2014_2080_MOESM1_ESM.pptx]

## Slide 1
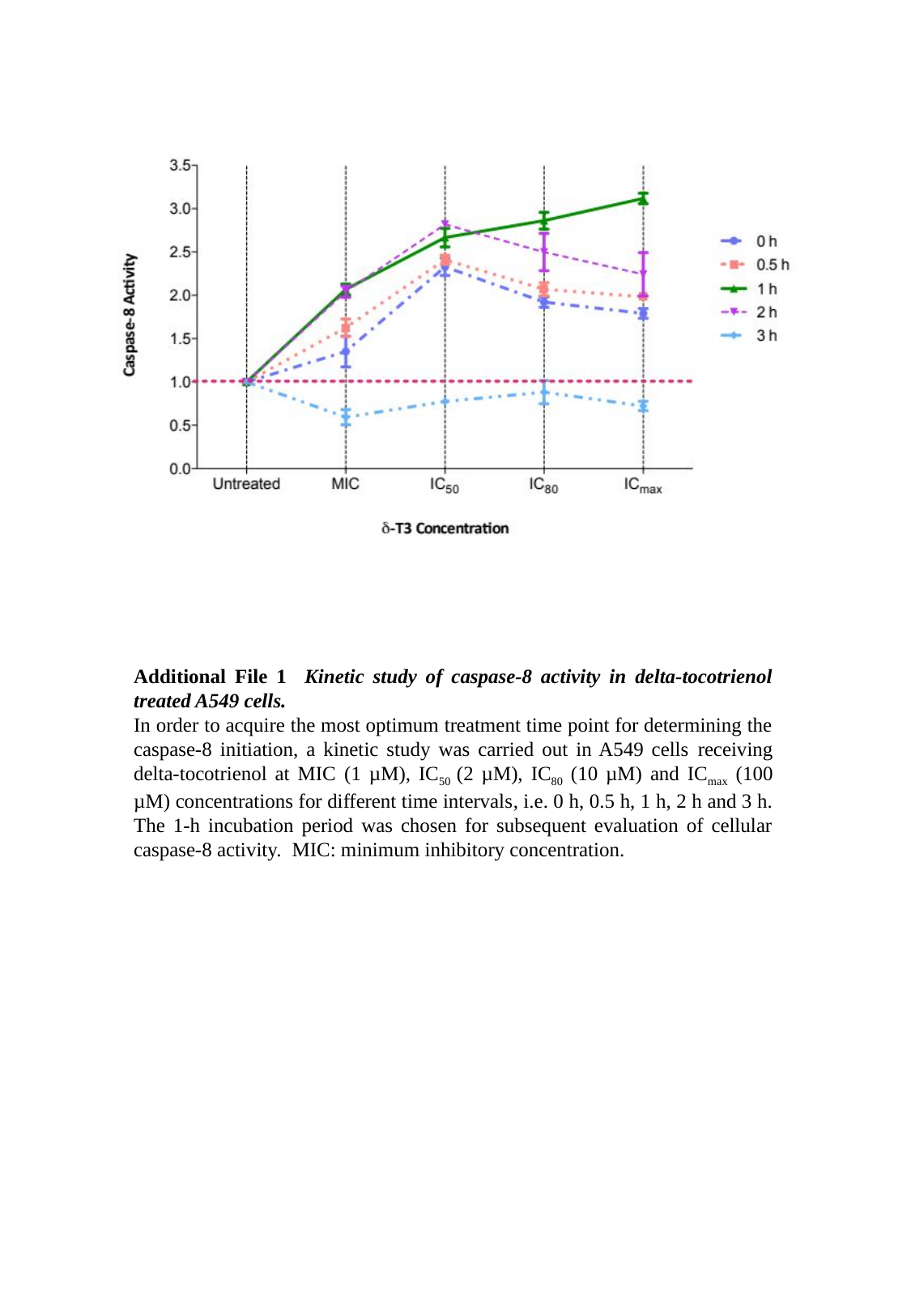

Additional File 1 Kinetic study of caspase-8 activity in delta-tocotrienol treated A549 cells.
In order to acquire the most optimum treatment time point for determining the caspase-8 initiation, a kinetic study was carried out in A549 cells receiving delta-tocotrienol at MIC (1 µM), IC50 (2 µM), IC80 (10 µM) and ICmax (100 µM) concentrations for different time intervals, i.e. 0 h, 0.5 h, 1 h, 2 h and 3 h. The 1-h incubation period was chosen for subsequent evaluation of cellular caspase-8 activity. MIC: minimum inhibitory concentration.
