# Supplementary material for: Cytotoxicity and apoptotic activities of alpha-, gamma- and delta-tocotrienol isomers on human cancer cells
Source: BMC Complement Altern Med. 2014 Dec 6;14:469. doi: 10.1186/1472-6882-14-469 (PMC4295404; doi:10.1186/1472-6882-14-469)
Supplement: Supplementary file 2 — Additional file 2: Proposed apoptotic pathway. Proposed apoptotic pathway induced by alpha-, gamma- and delta-tocotrienols. Tocotrienol isomers are proposed to induce apoptosis in A549 and U87MG cell lines with the involvement of cross-talk between extrinsic and intrinsic pathways based on the evidences gathered from caspase-8-dependent cleavage of Bid which led to Bax activation, mitochondrial membrane potential loss and eventually cytochrome c release, hence resulting the initiation of downstream effector caspases. (PPTX 68 KB) [file 12906_2014_2080_MOESM2_ESM.pptx]

## Slide 1
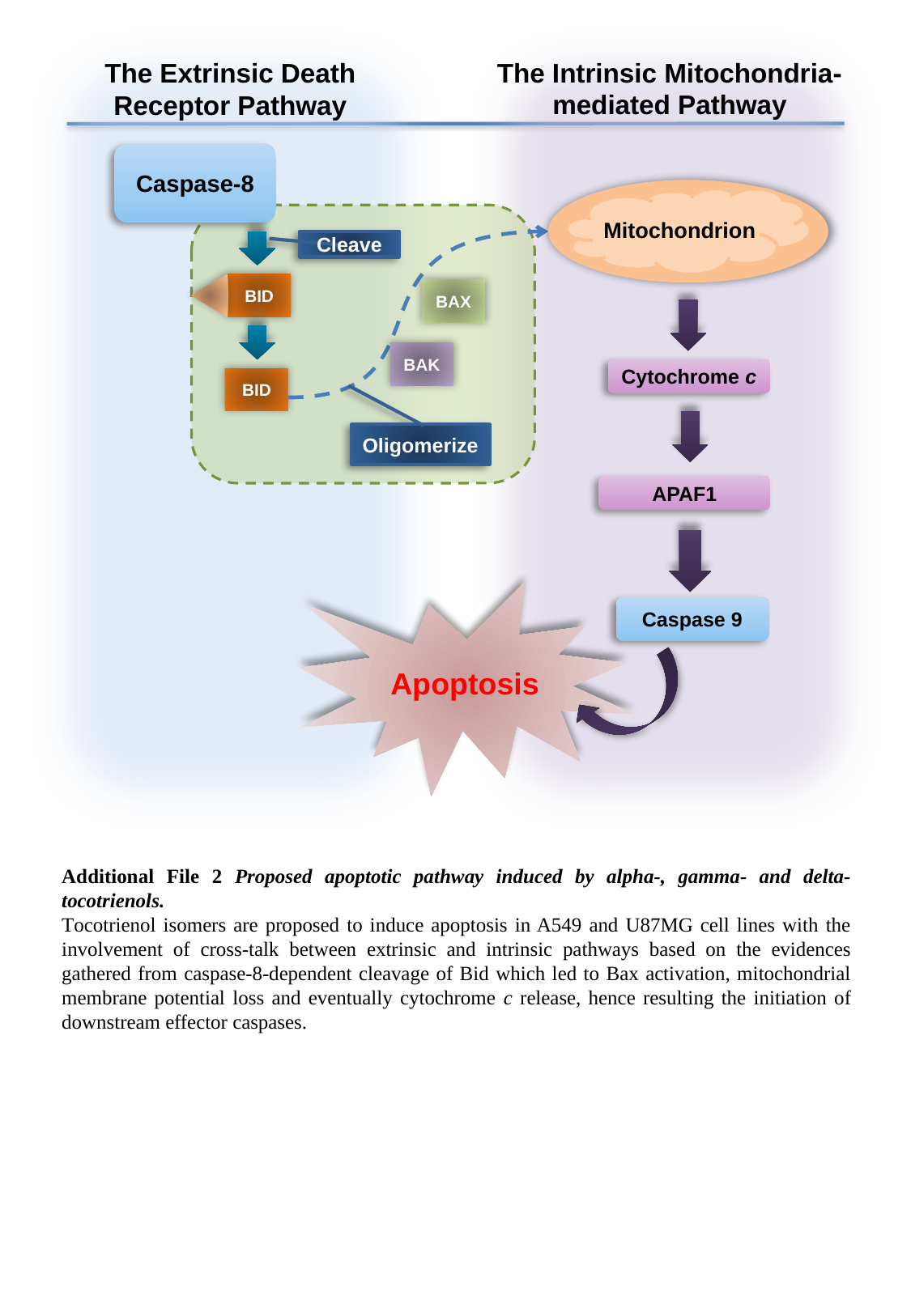

The Intrinsic Mitochondria-mediated Pathway
The Extrinsic Death Receptor Pathway
Caspase-8
Mitochondrion
Cleave
BID
BAX
BAK
Cytochrome c
BID
Oligomerize
APAF1
Apoptosis
Caspase 9
Additional File 2 Proposed apoptotic pathway induced by alpha-, gamma- and delta-tocotrienols.
Tocotrienol isomers are proposed to induce apoptosis in A549 and U87MG cell lines with the involvement of cross-talk between extrinsic and intrinsic pathways based on the evidences gathered from caspase-8-dependent cleavage of Bid which led to Bax activation, mitochondrial membrane potential loss and eventually cytochrome c release, hence resulting the initiation of downstream effector caspases.
